# Linac-Based Radiosurgery for Patients With Brain Oligometastases From a Breast Primary, in the Trastuzumab Era-Impact of Tumor Phenotype and Prescribed SRS Dose

**DOI:** 10.3389/fonc.2019.00377

**Published:** 2019-05-28

**Authors:** Kevin Armstrong, Jennifer Ward, Mary Dunne, Luke Rock, Jennifer Westrup, Christopher R. Mascott, Pierre Thirion, Alina Mihaela Mihai

**Affiliations:** ^1^Beacon Hospital, Dublin, Ireland; ^2^Saint Luke's Radiation Oncology Network, Dublin, Ireland; ^3^Department of Radiation Oncology, Beacon Hospital, Dublin, Ireland; ^4^Department of Medical Oncology, Beacon Hospital, Dublin, Ireland; ^5^Medical Center Bergan, Creighton University, Omaha, NE, United States

**Keywords:** brain metastases, SRS, Her 2 status, breast cancer, dose

## Abstract

**Background:** The role of stereotactic radiosurgery (SRS) in the treatment of limited numbers of brain metastases in selected breast cancer patients is well-established.

**Aims:** To analyse outcome from a single institutional experience with SRS, to identify any significant prognostic factors and to assess the influence of Her-2, estrogen receptor status, and prescribed dose on outcome.

**Methods:** The medical records of 56 patients treated at in a single institution between 2009 and 2014 were reviewed. Demographic, treatment related and outcome data were analyzed to identify prognostic factors in this patient population. The primary endpoints were overall survival and local control. Secondary endpoint was distant intra-cranial progression-free survival.

**Results:** The median follow- up time for the entire cohort was 10.33 months (1.25–97.28). The overall median survival was 12.5months (95%CI = 5.8–19.2), with 53.3%, and 35.8% surviving at 1- and 2- years post-SRS. After adjustment for the effect of Her 2 status, uncontrolled extra-cranial disease at the time of SRS predicted for shorter survival (HR for death = 3.1, 95% CI = 1.4–6.9, *p* = 0.006). At the time of death, 75% of the patients had active, uncontrolled intra-cranial disease, with 56% these patients presenting intra-cranial disease only. Sustained local control was observed in 56 (59.6%) of 94 treated metastases. In univariate analysis, Her2 status, ERHer2 group status?, and prescribed SRS dose were highly significant for local progression free-survival (LPFS). After adjustment for the effect of Her 2 status, patients receiving 12–16 Gy can expect shorter LPFS than those receiving 18–20 Gy (HR = 1.7, 95% CI = 1.0–2.8, *p* = 0.043). After adjustment for the effect of dose group, patients with Her 2 negative cancer can expect shorter LPFS than those with Her 2 positive cancer (HR = 2.6, 95% CI = 1.5–4.4, *p* < 0.0005). Use of prior WBRT did not impact survival, local or distant intra-cranial progression-free survival.

**Conclusions:** Survival outcome is similar to the published literature. Improved outcomes are observed in patients with Her 2-positive, controlled extracranial disease at the time of SRS and higher SRS dose delivered. Achieving intra-cranial control appears to be an important factor for the survival of the breast cancer patients in the era of targeted therapies.

## Introduction

Brain Metastases occur in 20–40% of patients with metastatic cancer (1). Whole brain radiotherapy (WBRT) and steroid therapy have historically been used as the standard management. However, outcome is poor with this approach (2). Corticosteroid treatment has a modest impact, extending median survival by as little as 1–2 months and has significant toxicities. WBRT has a greater impact, but median survival is still measured in months (3). The biggest disadvantage with WBRT is that it doesn't result in a high prolonged local control rate, which contributes to overall low survival. Due to this limitation of WBRT investigators explored the use of surgical removal of oligometastatic (limited number) brain metastases in selected patients. In a randomized trial, Patchell et al. reported a median survival of 19 months in patients treated for solitary brain metastases, with surgical resection and WBRT compared to 9 months in those treated with WBRT alone (4). This trial which included patients with breast and other primary sites demonstrated the potential value of aggressive local intervention for oligometastatic brain tumors in patients with good performance status and controlled extracranial disease.

Historically stereotactic radiosurgery (SRS) was pioneered by Leksell for managing intracranial conditions such as arteriovenous malformations (5, 6) Following its successful use for benign brain conditions the technology was applied to brain metastases with results similar to those reported for surgery (7). SRS offers a non-invasive treatment alternative, which is performed as an outpatient procedure and generally well-tolerated.

As the systemic treatment of metastatic breast cancer has evolved and improved the prospect of achieving durable control of extracranial disease has increased dramatically. This has created a greater demand for the successful treatment of brain metastases of breast cancer patients for two reasons. Firstly, more patients fulfill the selection criteria by virtue of the control of extracranial disease and the fact that their performance status is higher systemic therapies are increasingly better tolerated. The second main reason is the identification of Her-2-Neu positive breast cancer. Up to 30% of breast cancer patients overexpress the Her-2-Neu receptor (8). This overexpression is associated with an aggressive phenotype. However, with the discovery of Trastuzumab, a monoclonal antibody against Her-2-Neu the prognosis has dramatically improved. In the metastatic setting, up to 30 to 40% of such patients will ultimately develop brain metastasis. The reason for the high incidence of brain metastasis in Trastuzumab treated patients is assumed to be because Trastuzumab (which is a large monoclonal antibody) may not cross the blood-brain barrier. As more patients achieve control of their Her-2-Neu positive extracranial disease they may develop brain metastases in a setting where SRS is clinically appropriate. It is therefore important to assess which prognostic factors will affect the outcome of this therapy and to define an optimal dose range.

## Materials and Methods

The study was approved by the local Institutional Review Board.

### Cohort

A retrospective analysis was performed on 56 patients with metastatic breast cancer with metastases to the brain. All of the patients were treated with Stereotactic Radiosurgery (SRS) or intensity modulated radiosurgery (IMRS) between 2009 and 2014. All patients had a fine resolution 3DMRI of the brain (confirming diagnosis of brain metastases) within the 14 days preceding the treatment.

An Excel database was generated which included the patients demographics (age, date of diagnosis, treatment, pathology, brain progression details), treatment related and outcome related data. The Disease-specific graded prognostic assessment score (DS-GPA) was retrospectively calculated in all patients. DS-GPA score is a prognostic scoring system specifically designed for brain metastases. It takes account of performance status, age, number of brain metastases, and status of extracranial disease to assign a class ranging from I-IV. Class I has the best prognosis. Information was gathered on these patients using the hospital's electronic (ARIA) and paper charts.

### Planning Technique

For all patients a dedicated contrast-enhanced planning brain CT was acquired, with slice thickness of 1.25 mm, using the frame or frameless systems for localization of the lesions. Of the 56 patients receiving SRS, 37 had a frameless mouth-bite coordinate system applied to minimize patient discomfort; 19 patients had a frame-based coordinate system attached under local anesthesia due to inadequate dentition required for the frameless system.

The planning CT was co-registered with fine resolution brain MRIs (T1, T2, SPGR, FLAIR sequences with and without contrast). The use of contrast for the planning CT can help to identify small structures such as blood vessels which can be cross referenced on the planning CT and the fused MRI to assess the accuracy of image fusion. This is particularly relevant when the metastasis is not visible on the CT and target volume definition is reliant on the fused MRI. The gross tumor volume (GTV) was defined as the enhancing lesion on the CT and/or MRI T1 SPGR contrast enhanced sequence. The planning target volume (PTV) was defined as the GTV with a 1 mm circumferential margin. Varian Eclipse treatment planning system (Varian, Palo Alto, CA) was used to generate cone-based SRS or intensity modulated radiosurgery (IMRS) plans. For tumors < 3 cm maximum dimension in any plan, a single fraction of 14–24 Gy was delivered, generally using the RTOG guidelines (9). The variation in the doses prescribed, not conforming with these ranges, was due to individual physician choice, particularly when lower doses were used. For larger tumors, IMRS was used to deliver either 30 Gy/5 fx or 24 Gy/3 fx. The dose was prescribed at the 80% isodose line for cone-based SRS, while a minimum isodose of 95% prescription dose covered the target for the IMRS plans. Treatment characteristics for the 56 patient included in this study are depicted in Table 1.

**Table 1:** Treatment characteristics for 94 treated brain metastases in 56 patients with primary breast cancer.

**Parameter**		
Number of brain metastases treated/patient	1	33 pts (58.9%)
	2–3	18 pts (32.2%)
	4–5	5 pts (8.9 %)
Tumor size (mm)		
	Mean ± STDEV	17.6 ± 8.5 mm
	Median (Range)	16 (3–40)
Dose fractionation	21–24 Gy/1 fx	12 lesions (12.8%)
	18–20 Gy/1 fx	38 lesions (40.4%)
	14–16 Gy/1 fx	37 lesions (39.4%)
	< 14 Gy/1 fx	2 lesions (2.1%)
	30 Gy/5 fx or 24 Gy/3 fx	5 lesions (5.3%)

*pts, patients; fx, fraction*.

The SRS/IMRS was delivered using a Varian Trilogy Tx linear accelerator, using a cone-based or MLC based technique. Stereotactic localization was provided using the Varian SonArray infra-red localization or Vision RT surface guidance (from 2014 onwards) systems.

Steroids were not routinely recommended, however patient on steroids at the time of SRS (22 patients) were kept on the same dose (no modifications) during treatment.

### Follow-Up

Follow-up data were collected from institutional records, records from referring facilities and family physicians. After SRS, patients generally underwent routine follow-up clinical examination and imaging. MRI brain (as described above) was performed at 2 months' post SRS, then every 3 months for the first 2 years. In case of suspicion of pseudoprogression, a short-interval (6–8 weeks) MRI brain was done. For patients unable to attend our institution for follow-up, the data was retrieved from other institutional or family physician records. The data was reviewed and the response and reported toxicity were scored retrospectively. MRI images were routinely reviewed by a neurosurgeon with expertise in imaging neuroanatomy.

### Statistical Analysis

The primary endpoints were local control and overall survival. The secondary endpoint was distant intra-cranial progression-free survival.

Local control was defined as stability or reduction in size of the treated lesion(s) on serial MRIs. MRI response was analyzed by a neurosurgeon with expertise in brain MRI response assessment. Distant intracranial progression was defined as development of new lesion(s) outside the treated metastasis.

Patient, tumor and treatment characteristics were summarized. The following factors were analyzed for impact on local, and distant intra-cranial progression-free survival: age, clinical presentation (symptomatic vs. incidental), GPA, status of the extracranial disease at the time of SRS (controlled yes vs. no), ER status (positive vs. negative), Her 2 status (positive vs. negative), location of brain metastases (supra vs. infratentorial), number of brain metastases (targets: 1 vs. 2–3 vs. 4–5), lesion size (as a continuous variable), dose prescribed (12–16 vs. 18–20 vs. 20–24 vs. IMRS), time to development of brain metastases from the initial diagnosis (< 1 year or >1 year), and WBRT (yes vs. no).

Categorical variables were analyzed using chi-square tests. The Kaplan-Meier method was used to estimate survival times, and the log -rank tests to compare differences in survival. Survival was calculated from the date of SRS/IMRS to the date last follow-up/ death (overall survival, OS), to the date of first local progression/ death (local progression-free survival, LPFS) or to the date of first distant progression/ death (distant progression-free survival, DPFS). Overall and distant intra-cranial progression-free survival were analyzed by individual patient, while the local progression-free survival was analyzed by individual metastasis. The Cox proportional hazards model was used to assess the effects of co-variates (statistically significant in univariate analysis) on survival. All statistical tests were two-sided and assessed for a significance at 0.05 level. Statistical analyses were carried out using IMB SPSS statistical program version 24.

## Results

### Cohort

The cohort included 56 females with brain metastases from a breast cancer primary, with a median age of 52.8 years (30.8–82.5). Patient, tumor and treatment characteristics are detailed in Table 2. The majority of the patients (*n* = 54, 96.4%) had a Karnofsky performance status score (KPS) >70 and GPA of at least 2 was recorded for 68% of the patient Most patients (*n* = 35, 62.5%) had either no or controlled extra-cranial disease at the time of SRS, 70% of them received prior chemotherapy and 50% received systemic concurrent treatments (Herceptin or Taxanes).

**Table 2:** Demographics, treatment and target characteristics in 56 patients with brain metastases from a breast cancer primary, who received stereotactic radiosurgery between 2009 and 2015.

		**Number (%)**
Age	Mean ± STDEV	53.1yo ± 12.0
	Median (Range)	52.8 (30.8–82.5)
Gender	Males	0 (0%)
	Females	56 (100%)
KPS	60	2 (4%)
	70	11 (20%)
	80	27 (48%)
	90	16 (29%)
GPA	1	12 (21%)
	2	24 (43%)
	3	14 (25%)
	Unknown	6 (11%)
Extracranial disease controlled at the time of SRS	No	16 (29%)
	Yes	35 (62%)
	Unknown	5 (9%)
Her 2 status	Positive	33 (59%)
	Negative	20 (36%)
	Unknown	3 (5%)
ER status	Positive	29 (52%)
	Negative	24 (43%)
	unknown	3 (5%)
Prior chemotherapy	Yes	40 (71%)
	No	16 (29%)
Concurrent systemic treatments (herceptine, hormones)	Yes	28 (50%)
	No	26 (46%)
	Unknown	2 (4%)
Time interval between initial diagnosis and BM(months)	Mean ± STDEV	57.4 ± 43.6
	Median (Range)	44.0 (2.8–220.8)
Presentation	Incidental finding	22 (39%)
	Seizures	2 (4%)
	Headaches	12 (21%)
	Other neurological symptoms	20 (36%)
SRS intent	At progression after WBRT	24 (43%)
	Boost after WBRT	10 (18%)
	Boost after resection	2 (4%)
	Alone	20 (36%)
No intracranial metastases at the time of SRS	1	33 (59%)
	2	16 (29%)
	3	2 (4%)
	4	3 (5%)
	5	2 (4%)

The average age at the time of development of brain metastases was 52 years old (30–82). The median time from initial diagnosis to development of brain metastases (BM) was 44.04 months (2.82–220.8) and the median time from initial diagnosis to the SRS was 51.6 months (3.15–221.7). The average time to development of BM was significantly longer in patients with ER+ disease (ER+ vs. ER– = 76.7 vs. 32.2 months, *p* = 0.0001). Her 2 negative status was associated with longer time to development of BM, but it did not reach significance (Her 2– vs. Her 2+ = 69.6 vs. 47.7, *p* = 0.07).

Most of the patients (*n* = 33, 58.9%) were treated for a single brain metastasis. The median tumor size was 16 mm (3–40). However, there were five patients with more than five brain metastases at the time of SRS (one patient with six lesions, one with seven, and three patients with eight); for these patients only the progressing lesions (after prior WBRT) received SRS.

### Survival

The median follow- up time for the entire cohort was 10.33 months (1.25–97.28). At the time of the last known follow-up, 17 patients (30.4%) were alive, and 39 (69.6%) have died. Among the 39 patients who died, 29 (74.35%) had uncontrolled intra-cranial disease at the time of death (13 both intra and extracranial disease uncontrolled and 16 intracranial disease only).

**Figure 1:**
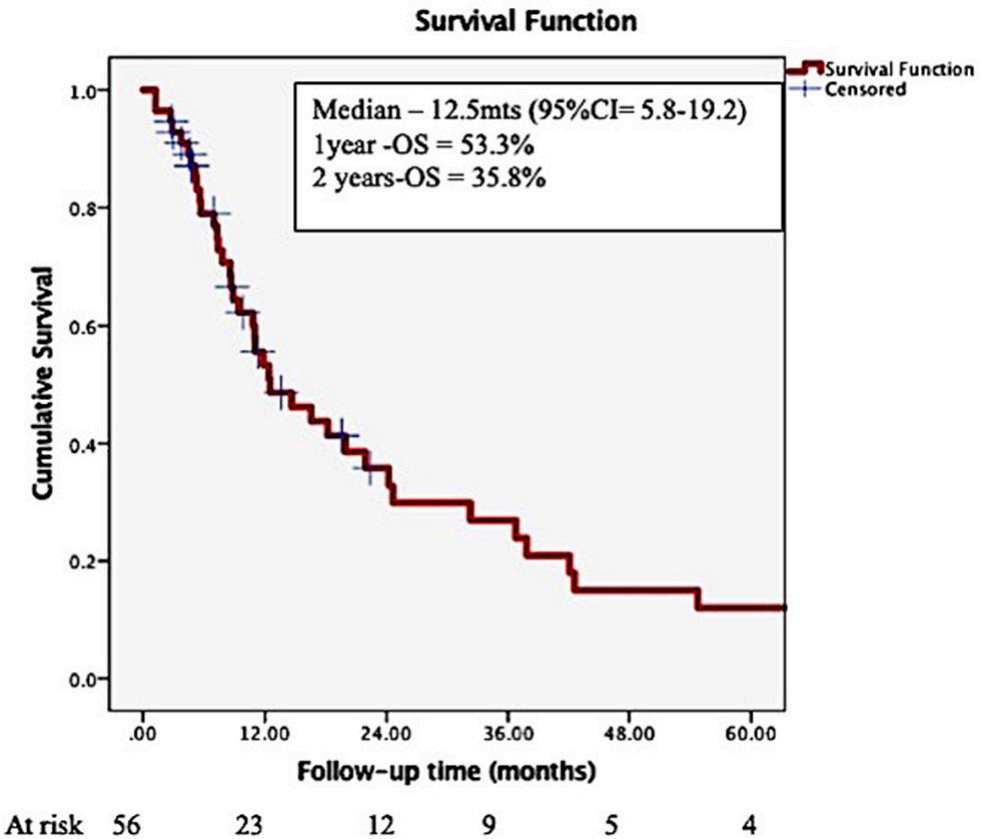
Overall survival in patients with brain metastases from a breast primary, treated by stereotactic radiosurgery.

The overall median survival was 12.5 months (95% CI = 5.8–19.2), with 53.3%, and 35.8% surviving at 1- and 2- years post-SRS (Figure 1), with a small proportion (5–20%) surviving more than 5 years after the initial SRS.

**Figure 2:**
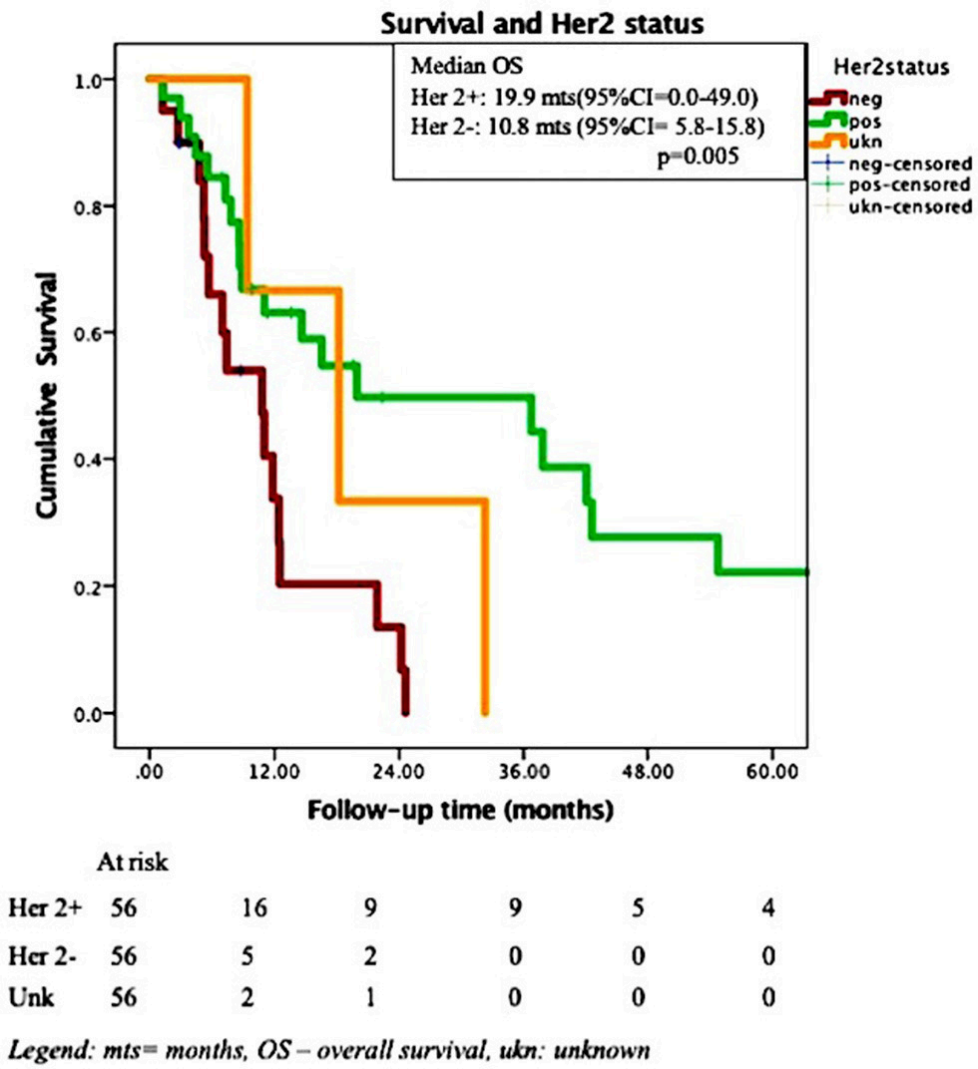
Overall survival and Her 2 status in 56 patients with brain metastases treated by SRS.

**Figure 3:**
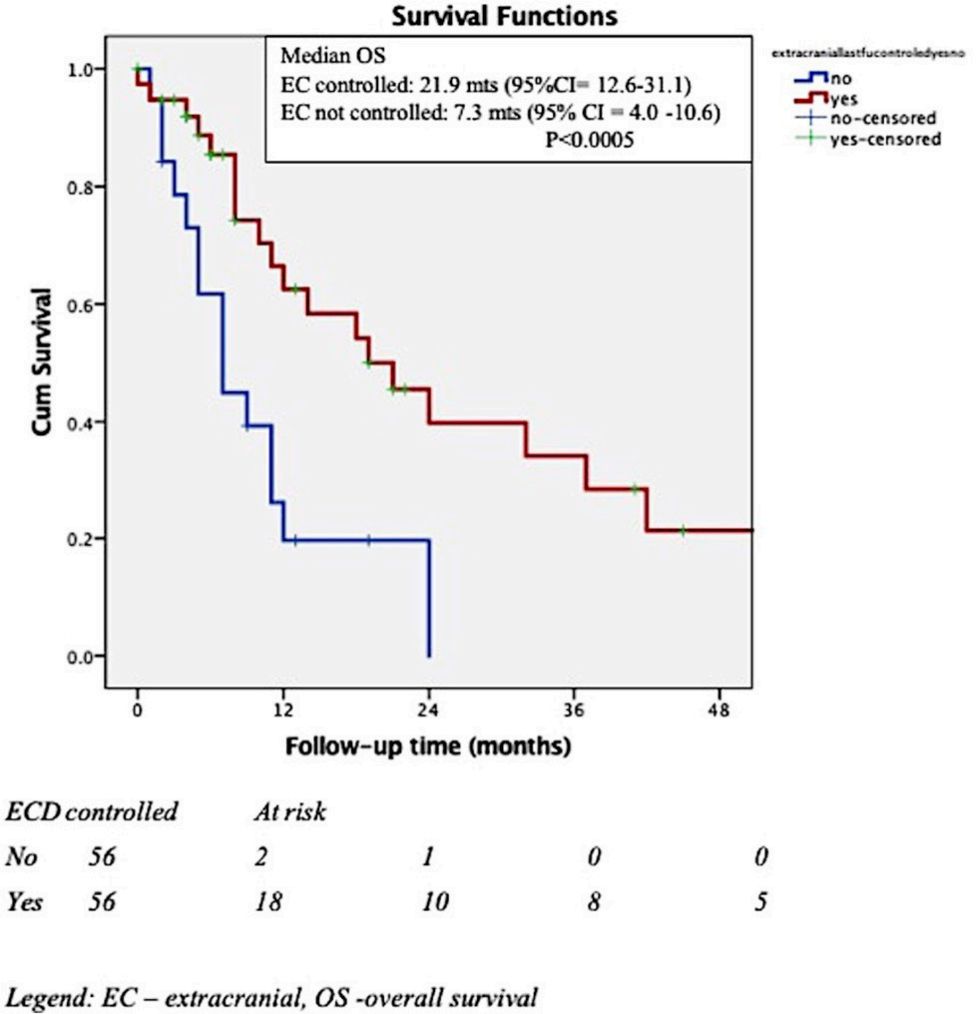
Overall survival and extracranial (EC) control.

In Cox multivariate analysis (MVA), after adjustment for the effect of Her 2 status, controlled extra-cranial disease at the time of SRS (HR for death if uncontrolled ECD = 2.9, 95% CI = 1.3–6.3, *p* = 0.009) was significantly associated with OS. The Her 2 status presented a trend toward significance (HR for death for Her 2 negative cancer = 2.1, 95% CI = 0.97–4.9, *p* = 0.057) after adjustment for the effect of extra-cranial disease. Addition of whole brain RT (WBRT) was not associated with increased OS. Figures 2, 3 depict the OS function of the Her 2 status and the status of extracranial disease.

### Local Control

During follow-up, 35 lesions (37%) have progressed, after a median of 7.3 months (1.25–97.28). Six lesions were salvaged by further local treatments (1-surgery, 2-IMRS, 3-SRS). Therefore, at the last known follow-up, of the 94 treated lesions, 61 (64.9%) were controlled locally, 29 progressed (30.9%) and we were unable to assess the response for 4 lesions (4.5%).

**Figure 4:**
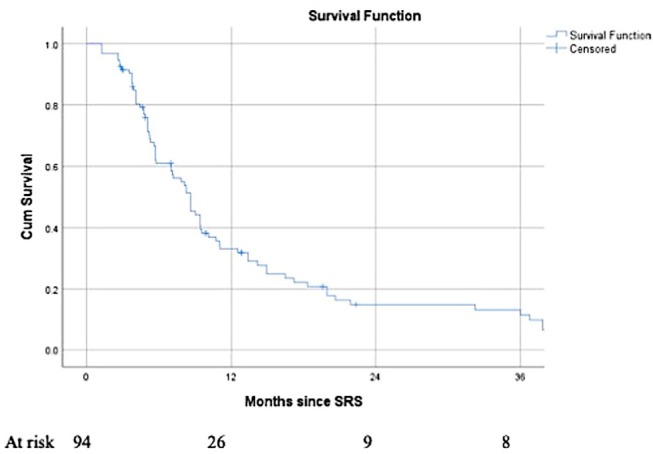
Local Progression-free survival for 94 brain metastases from a breast primary, treated by SRS.

The median LPFS was 8.6 months (7.0–10.2), with 1- and 2 years-LPFS of 33 and 15%, respectively. LPFS is depicted in Figure 4. In univariate analysis, Her 2 status, ERHer2 group, and dose group were highly significant for LPFS (Table 3). After adjustment for the effect of Her 2 status, patients receiving 12–16 Gy can expect shorter LPFS than those receiving 18–20 Gy (HR = 1.7, 95% CI = 1.0–2.8, *p* = 0.043). After adjustment for the effect of dose group, patients with Her 2 negative cancer can expect shorter LPFS than those with Her 2 positive cancer (HR = 2.6, 95% CI = 1.5–4.4, *p* < 0.0005). Use of WBRT did not impact LC. Table 4 presents the local recurrence rates for small lesions (< 2cm) function of tumor size and dose received. Table 5 presents the local recurrence rates for all treated lesions, function of the dose received.

**Table 3:** Univariate analysis variables significant for local progression free survival in a cohort of 56 breast cancer patients with 94 brain metastases, treated by SRS.

		**Estimated median LPFS (mts)**	**95% CI**		***p*-value** ** (log-rank)**
			**Lower bound**	**Upper bound**	
Her 2	**Positive**	**10.1**	7.4	12.8	< 0.0005
	Negative	5.7	5.2	6.1	
ERHer2group	**ER+Her2+**	**10.7**	8.1	13.4	0.001
	ER−Her2+	9.528	7.7	11.3	
	ER+Her2−	5.1	4.7	5.4	
	ER−Her2−	5.7	5.4	6.0	
SRS dose prescribed	12–16 Gy/1 fx	7.1	3.8	10.4	0.006
	18–20 Gy/1 fx	8.6	7.6	9.6	
	**21–24 Gy/1 fx**	**9.4**	4.5	14.3	
	IMRS (24–30 Gy/3 fx)	3.9	1.9	5.9	

*ER, estrogen receptor; fx, fraction; LPFS, local progression-free survival; mts, months*.

**Table 4:** Local progression rates for 64 small lesions (< 2cm) according to the tumor size and SRS dose prescribed.

			**Local progression**		
			**Yes**	**No**	**Total**
Size-dose	TS < 1 cm	Count	**1**	6	7
group	22–24 Gy/1 fx	% within group	**11.1%**	66.7%	100.0%
	TS < 1 cm	Count	**4**	13	17
	18–20 Gy/1 fx	% within group	**23.5%**	76.5%	100.0%
	TS < 1 cm	Count	**3**	2	5
	12–16 Gy/1 fx	% within group	**60.0%**	40.0%	100.0%
	TS 1.1–2 cm	Count	**0**	3	3
	22–24 Gy/1 fx	% within group	**0.0%**	100.0%	100.0%
	TS 1.1–2 cm	Count	**3**	12	15
	18–20 Gy/1 fx	% within group	**18.8%**	75.0%	100.0%
	TS 1.1–2 cm	Count	**9**	8	17
	12–16 Gy/1 fx	% within group	**52.9%**	47.1%	100.0%
Total		Count	20	44	64
		% within group	29.9%	65.7%	100.0%

Lesions for which the response was not known were excluded.

*TS, tumor size (diameter). Chi-square test not valid because of small numbers in some cells*.

**Table 5:** Local progression rates for 90 treated lesions, according to the SRS dose prescribed.

**Dose group**		**Local Progression**		**Total**
		**No**	**Yes**	
12–16 Gy/1 fx	Count	17	**21**	38
	% within dose group	44.7%	55.3%	100.0%
18–20 Gy/1 fx	Count	27	**10**	37
	% within dose group	73.0%	27.0%	100.0%
22 Gy/1 fx	Count	9	**1**	10
	% within dose group	90%	10%	100.0%
24–30/3–5 fx	Count	2	**3**	5
	% within dose group	40.0%	60.0%	100.0%
Total	Count	55	35	**90**
	% within dose group	61%	39%	100.0%

Lesions for which the response was not known were excluded.

*Chi-square test not valid because of small numbers in some cells*.

### Distant Intra-cranial Progression-Free Survival

During follow-up, 23 patients (41%) developed distant intra-cranial progression. The median DPFS was 9.85 months (7.6–12.1), with actuarial 1-, 2-years DPFS of 40.1 and 11.8%, respectively. None of the variables analyzed was significantly associated with DPFS. Particularly, WBRT either prior to, at the time to SRS or at progression, did not affect DPFS.

### Toxicity

There was no G3 or more acute or late toxicity identified for this cohort. The most commonly identified side effect was fatigue grade 1-2, in 10 patients (17.8%).

## Discussion

This experience from a single institution confirms some of the findings reported from other series. The median survival of 12 months is in keeping with other publications. In this series, survival was 53.3% at 1 year after SRS and 35.8% at 2 years. Kondziolka et al. from UPMC reported the outcome for 350 breast cancer patients with 1535 brain metastases (10). Overall survival was 49% at 1 year, 26% at 2 years with a median survival of 11.2 months.

In multiple series reporting SRS for brain metastases, the primary site of origin is an important predictor of outcome (11–13). Breast cancer origin appears to be associated with improved overall survival compared to other histologies (12). It may also predict a higher prospects of achieving local control of the treated metastases. Results of several retrospective studies in patients with brain metastases from a breast primary, treated by SRS, are presented in Table 6. These series (10, 14–28) identified several factors which impact on the outcomes of these patients, with longer survival reported for higher KPS, lower RPA class, single small metastasis (< 1 cm), deep cerebral location, controlled extracranial disease and ER+ or Her2 + the biological subtypes. In this series we focused on outcome for breast cancer patients only. We observed that patients with Her-2+ brain metastases developed the metastases sooner after diagnosis than other phenotypes, in line with prior publications. This may reflect the more aggressive natural history of this subtype and its particular predilection for brain spread. Similarly, estrogen negative patients developed their brain metastases after diagnosis sooner than estrogen positive patients. However, on MVA, after controlling for other variables, the only factor associated with improved OS was controlled extra-cranial disease at the time of SRS. After adjustment for the effect of Her 2 status, LPFS was significantly correlated with the SRS dose group. After adjustment for the effect of dose group, Her 2 status was highly predictive for LPFS patients (with Her 2–status is associated with shorter LPFS with Her 2 +, HR = 2.6, 95% CI = 1.5–4.4, *p* < 0.0005). None of the other factors analyzed had predictive value for the studied outcomes.

**Table 6:** Selected studies of brain SRS in patients with brain metastases from a breast primar.

**Study**	**No patients/no lesions/dose**	**Survival**	**Local and intra-cranial control**	**Observations**
Shenker et al. (14)	128 pts 1-2BM/pt 20Gy/fx (10-24Gy)	- medOS-16.3 mts OS-1y = 56% OS-2y = 18% OS-2y = 10%	- IC failure−6 m = 24% - IC failure−12 m = 41% - IC failure−24 m = 51%	- ER,PR ± trend toward decreased neurological death - Factors associated with non-neurological death: status extracranial disease, dose, Her 2 status
Wolf et al. (15)	200 pts 1237 BM (diff histology) Med 18Gy/fx		LC1y = 97% LC2y = 93% LC = 100% for TS < 1 cm	Increased survival for lesions < 1 cm
Pessina et al. (16)	66 pts Surgery–SRS/WBRT	Med OS = 30.7 mts OS-1y = 78.5% OS-2y = 57.4% OS-3y = 43.3%	LRR-24.2% LC-1y = 87.5% LC-2y = 71.2% LC-3y = 63%	- Factors associated with survival: KPS, number of BM, local treatment performed, status of EC disease at the time of dg of BM, treat with Herceptine
Mix et al. (17)	214 pts 23% GK SRS 46% SRS-WBRT 31% WBRT	Med OS21 mts SRS vs. 3 mts WBRT	NR	- WBRT prior or as salvage did not impact survival - Tumor volume and Her 2 status significantly associated with OS - ER status did not impact on OS
Roehrig et al. (18)	111 pts	Med OS = 16.8mts OS-1y = 59.5% OS-2y = 38.4%	NR	KPS – strongest predictor for survival in MVANo impact of number lesions, WBRT
Mohammadi et al. (19)	896 pts- 3034BM (< 2 cm in size) 166 breast cancer	Med OS = 14.9 mts	- New IC lesions rate-45% after a median of 10.2 mts - 10% rate of local progression	- Factors associated with local/IC control: tumor diameter (< or >1cm), tumor volume, conformality index, prescribed dose (24Gy vs. < 24)
Nieder et al. (20)	25 pts brain -only mets WBRT+/-SRS	MedOS−11.7 mts OS-1y = 48% OS- 2y = 28%	Brain PFS - Med = 6.2 mts - @1y = 22% Med time to brain progression−10.8mts Freedom of brain progression @1y−36%	- Predictors for OS: KPS, TNBC, coordination deficits, lack of upfront surgery, lack of hormone therapy/herceptine - Predictors for brain PFS: KPS, location (cerebellar worse), cognitive or coordination deficits, systemic treatments after SRS
Cho et al. (21)	131 pts Med−3 lesions/pt (1-22)	- Med time SRS to death = 15.7 mts - Med OS = 7 mts for TNBC		- ER+Her2- and Her 2 + - longest survival - TNBC poor prognostic - Prior WBRT, age – no impact - Cerebellar lesions TNBC – worse survival
Yang et al. (22)	136 pts 186 BM	Med Sv- 17.6 mts OS-1y = 65% OS-2y = 45%	LF-1y = 10% Regional failure @12mts = 45%	- In MVA – predictors for Sv: >1lesion, TNBC, active EC disease - EC disease associated with regional failure - Tumor size – associated with risk of LF
Tam et al. (23)	57pts 28pts Her2+	Her 2+ vs. Her 2- Med OS = 22 vs. 12 mts	Her2+ vs. Her 2- - medTTP- 7 vs. 11mts - Salvage tt: 50% vs. 21%	- Her 2+ appears to show higher rates of intra-cranial relapse, despite better OS rates
Yomo et al. (24)	80 pts 40 pts Her 2+	Lapatinib vs. non-lapatinib tt: -OS-1y = 50% vs. -OS−2 y = 26%	LC−1y = 84% LC−2yc = 70% Lapatinib vs. non-lapatinib LC-1y = 86 vs. 69%	- Factors associated with survival: Her 2 status, RPA class, total PTV at initial SRS - Factors associated with local control: tumor volume, peripheral dose
Xu et al. (25)	103 pts – 24 with TNBC	TNBC vs. non-TNBC- OS (after dg): 43 vs. 82 mts - Neurological Sv: 13 vs. 25 mts - Radiosurgical Sv: 6 vs. 16 mts		- TNBC – adverse prognostic factor
Kelly et al. (26)	79 pts Had salvage SRS>3mts after initial treatment 76 of them - WBRT	Med OS = 9.8 mts	Brain PFS Median = 5.7 mts post-SRS	- Her 2+ status and stable EC disease have improved clinical course and survival - 82% of these patients would require further systemic treatment
Caballero et al. (27)	310 pts salvage SRS 90 pts – breast cancer	Med OS −8.4 mts		Favorable fact for survival in breast cancer patients: single brain met, age < 50, longer time interval WBRT-SRS
Kondziola et al. (10)	350 pts 1535BM SRS at dg or at recurrence Srs dose -RTOG criteria	OS 6mts-69% 12mts−49% 24 mts−26%		- Longer OS if controlled EC disease, lower RPA, higher KPS, smaller number of metastases, smaller tumor volume, deep metastases, Her 2+
Karam et al. (28)	441 pts 40% Her 2+	Med OS (from brain treat)-4.5 mts Med OS RPA 1vs. 2 vs. 3 = 14.5 vs. 6.4 vs. 1.8 mts		- RPA class significantly associated with survival

Patients with Her 2 disease treated by Trastuzumab are at particular risk of developing brain metastasis. In the metastatic setting, up to 30–40% of such patients ultimately develop brain metastasis: three of the five adjuvant trails of Trastuzumab reported brain metastasis following the treatment. 1.6% of these patients ultimately develop brain metastasis (29). The reason for the high incidence of brain metastasis in Trastuzumab treated patients has been assumed to be due to the fact that Trastuzumab with 185 kDa molecular weight may not be able to cross the blood-brain barrier (BBB). Therefore, the brain might be a sanctuary site for malignant cells. Dijkers et al. (30) have performed Her2neu staining whole body scintigraphy and have demonstrated that Trastuzumab can partially penetrate the BBB. Additionally, Stemmler et al. (31) measured Trastuzumab levels in CSF of Her2neu positive brain metastasis patients and found that these levels were increased if meningeal carcinomatosis was present or if the patient had received WBRT. Analyzing these two sets of data one may postulate that BBB may be disrupted by tumor spread or by WBRT. However, it may not be disrupted by the presence of cells in the brain and this may allow brain metastasis to develop before the disruption of the BBB occurs. In other words, the cells may establish themselves as significant micro-metastatic deposits or small macroscopic deposits before the BBB is sufficiently disrupted to allow Trastuzumab to potentially treat the metastases.

The good outcome following the treatment of good prognosis limited brain metastases in Her2 positive disease may be primarily a manifestation of the overall favorable biology and efficacy of systemic therapy. However, Her 2 positive disease may be more radiosensitive than other cancer subtypes. Liang et al. (32) demonstrated *in vitro* Trastuzumab enhanced radiation-induced apoptosis in breast cancer cells in a Her 2 level-dependent manner. They postulated that PI3/Akt pathway may be involved in this effect. In a metanalysis by Dahabreh et al. (29), the addition of adjuvant Trastuzumab to chemotherapy resulted in a lower risk for developing locoregional recurrence (data from 3 of the 5 trials, 6,752 patients: RR 0.58, 95% CI = 0.43–0.77, *p* = 0.0002). However, it is not clear if this is due to concurrent radiosensitization or independent Trastuzumab activity.

Neurological death is defined as death in the presence of active intracranial or leptomeningeal disease. In 2017, McTyre (33) reported that disease specific GPA, number of brain metastases, melanoma histology and SRS dose are predictive for neurological death. Targeted therapies appear to delay neurological death. Their results are based on the analysis of outcomes of 738 patients with brain metastases (different histologies) treated by upfront SRS; in 30.6% of them neurological death occurred, while 42% died of non-neurological causes. In 2018, Shenker (19) reported the outcomes of 128 breast cancer patients treated by SRS (median doze 20 Gy/1 fx) for 1-2 brain metastases. In their series, ER+PR+ status was associated with a trend toward decreased neurological death, while status of extra-cranial disease, SRS dose and Her 2 status were associated with the non-neurological death. In our series, 75% of the patients who died had active, uncontrolled intra-cranial disease, with 56% of these patients presenting intra-cranial disease only at the time of death. Therefore, achieving intra-cranial control appears to be an important factor for the survival of the breast cancer patients in the era of targeted therapies. Moreover, 10–20% of the patients included in this cohort survived more than 5 years (Figure 1), further emphasizing the importance of intra-cranial control for survival. We could not identify any statistically significant differences between the neurological and non-neurological death groups (results not shown); however, the number of patients having uncontrolled intracranial disease at the time of death was higher for lower SRS dose delivered: 51% if 12–16 Gy/1 fx vs. 27% if 18–20 Gy vs. 20% if 21–24 Gy/1 fx (p-0.21).

In addition to clinical factors, the UPMC series (10) suggest that higher tumor dose predicted progression free survival. In patients with brain metastases from a breast primary treated by SRS, the reported 1y-LC varies between 69 and 90% (Table 5), utilizing SRS doses between 15 and 24 Gy/1 fx. However, most of the reported studies included patients who received WBRT. Very few studies report the outcomes of the patients who received SRS alone. The impact of dose on the local control was reported by two other large studies published in 2018 and 2017 (24) on patients with brain metastases from different primaries (including breast). Our study is in agreement with these previously published data: lesions treated with 21–24 Gy had a local progression rate of 8.3 %, those treated with 18–20 Gy had 21%, while 43 and 60% of those treated with 12–16 Gy and IMRS, (respectively) developed local recurrence.

Several randomized trials have assessed the value of adding whole brain radiotherapy (WBRT) to SRS for patients with limited number of brain metastases from a variety of primary sources (34). None of these trials have demonstrated an overall survival advantage to adding WBRT. It is not routinely added to SRS because it has also been demonstrated that adding WBRT to SRS increases the risk of cognitive deterioration. The use of WBRT added to SRS does however result in reduced occurrence of local progression at the treated lesions compared to SRS alone and reduces the occurrence of new brain metastases in other parts of the brain. In the trial conducted by the Alliance for Clinical Trials in Oncology local control of the treated metastases was 73% with SRS alone vs. 90% when WBRT was added to SRS (35). Patients treated with SRS alone received 24 Gy in a single fraction if lesions were < 2.0 cm or 20 Gy if lesions were 2 to 2.9 cm in maximum diameter. The focus of debate surrounding the results of these trials has understandably been the implications for the use of WBRT. However, these trials demonstrate increased local control when WBRT is added to SRS. This raises the possibility that enhanced local control of metastases treated with SRS could be achieved if the dose of SRS was increased to an optimal level, rather than adding WBRT.

A systematic review of SRS for brain metastases (arising from various multiple primary sites) demonstrates the wide variety of fraction sizes in use (36). Across the series included in the review, the range was 10 to 30 Gy. The optimal dose for single fraction SRS has therefore clearly not been identified and adopted in clinical practice. RTOG 90-05 was a dose escalation trial of single fraction SRS dose (37). That trial identified the maximum tolerated doses to be 24, 18, and 15 Gy for tumors of ≤ 20 mm, 21–30 mm, and 31–40 mm maximum diameter. These doses have since been adopted in clinical practice for previously untreated lesions often without the addition of WBRT. However, in the RTOG trial, all patients had recurrent previously irradiated primary or metastatic brain tumors. Therefore, a dose escalation trial restricted to previously unirradiated lesions would likely identify higher maximum tolerated doses. Therefore, a need is identified to conduct trials to ascertain the optimal dose for SRS in previously unirradiated cases.

In January 2019, a search on clinicaltrials.gov identified two ongoing trial escalating the dose of single fraction SRS: NCT02390518 (38) clinical trial (run by University of Utah) includes patients with 1–5 brain metastases, for whom dose escalation is preview, based on the tumor diameter and volume: for tumors < 1 cm, and < 0.52 cc: dose will be escalated to 26 Gy/1 fx, then 28 Gy/1 fx and finally to 30 Gy/1 fx. For tumors with diameters of 11–20 mm and volume 0.52–4.1 cc, dose will be escalated to 26 Gy/1 fx, then 28 Gy/1 fx and 30 Gy/fx. For large metastases with a diameters 21–30 mm and a volume 4.18–14.3 cc the dose will be escalated to 20 Gy/1 fx, then 22 Gy/1 fx and 24 Gy/1 fx. A second trial run by University of Texas (NCT02645487) (39) will escalate dose by 3 Gy/step, based on the tumor diameter: for metastasis ≤ 1 cm dose will be escalated from 24 Gy to 30 Gy/fx, for 1–2 cm size dose will escalate from 21 Gy to 27 Gy/fx; for metastases between 2 and 3 cm dose escalation from 18 to 24 Gy, and for large metastases (size 3–4 cm) dose will be escalated from 15 Gy to 21 Gy/fx.

The rapid ongoing evolution of systemic therapies targeting the individual phenotypic subtypes of breast cancer has implications for analyzing outcome of breast cancer brain SRS. The high risk of brain metastases in patients treated with the monoclonal antibody Trastuzumab, provides an illustration of how new systemic therapy can alter the risk of developing brain metastases (29, 40). It has long been recognized that systemic therapies can positively influence local control within the breast itself when radiation is used in breast conservation (33). New systemic therapies may also influence the radiosensitivity of brain metastases.

The limitations of this study are its small sample size and its retrospective nature. This analysis demonstrates encouraging results. The prolonged median survival and the moderate number of patients surviving for 2 to 3 years may be a significant advance compared to the outcome of treatment of brain metastases before SRS was developed. Further advances for such patients may result from a better understanding of biology, improved tailored systemic therapy, appropriate surgery and further developments in stereotactic radiosurgery itself.

## Conclusions

Survival outcome is similar to the published literature. Improved outcomes are observed in patients with Her 2-positive, controlled extracranial disease at the time of SRS and higher SRS dose delivered. Achieving intra-cranial control appears to be an important factor for the survival of the breast cancer patients in the era of targeted therapies.

## Ethics Statement

This is a retrospective study carried out in accordance with the recommendations of Beacon Hospital Ethics Research Committee. At the time of their treatment, all patients included in this study consented that their information can be used for the retrospective analysis of their outcomes. All subjects gave written informed consent in accordance with the Declaration of Helsinki. The protocol was approved by the Beacon Hospital Ethics Research Committee.

## Author Contributions

KA and JWa contributed to database creation, drafting of the article and editing. MD contributed to statistical analysis and editing. LR contributed to drafting of the article and editing. JWe, PT, and CM contributed to review draft and editing. AM contributed to project development, data analysis, review and editing of all versions of the draft. All authors gave final acceptance of the manuscript.

### Conflict of Interest Statement

The authors declare that the research was conducted in the absence of any commercial or financial relationships that could be construed as a potential conflict of interest.
